# Dynamic brain communication underlying face pareidolia in male schizophrenia

**DOI:** 10.1038/s41537-025-00656-4

**Published:** 2025-08-13

**Authors:** Valentina Romagnano, Julian Kubon, Alexander N. Sokolov, Andreas J. Fallgatter, Christoph Braun, Marina A. Pavlova

**Affiliations:** 1 Department of Psychiatry and Psychotherapy, Tübingen Center for Mental Health (TüCMH), Medical School and University Hospital, Eberhard Karls University of Tübingen, Tübingen, Germany; 2 German Center for Mental Health (DZPG), Partner Site Tübingen, Tübingen, Germany; 3 MEG Center, Medical School and University Hospital, Eberhard Karls University of Tübingen, Tübingen, Germany; 4 Hertie Institute for Clinical Brain Research, Medical School and University Hospital, Eberhard Karls University of Tübingen, Tübingen, Germany

**Keywords:** Schizophrenia, Neural circuits

## Abstract

Faces are essential for effective communication and social interaction. Substantial alterations in face processing are observed in a wide range of mental disorders, in particular, in schizophrenia (SZ). Individuals with SZ experience difficulties to seeing faces in face-pareidolia images that easily elicit face impression in their typically developing (TD) peers. Here, males with SZ and TD controls performed a task with Arcimboldo-like Face-n-Food face-pareidolia images during MEG recording. The outcome reveals that already at early processing stages, the bursts of gamma oscillations differ between SZ and TD individuals in terms of frequency and topography. When contrasting gamma activity for face responses between TD individuals and SZ, the maximum activation for the frequency range of 40–45 Hz originates from the right LOC. In accord with this, in SZ, an advanced analysis of brain connectivity unfolding over time in the low (40–45 Hz) and high (65–70 Hz) gamma ranges reveals alterations in communication between the right LOC and the social brain. In SZ, early engagement of the right LOC is limited to transmitting signals to higher-order regions, whereas in TD, it also serves as a recipient of sophisticated feedback communication from the higher-order areas of the social brain. This study offers novel insights into altered brain communication and the origins of social cognition deficits in SZ that is characterized by a skewed sex ratio with substantial gender differences in disease manifestation.

## Introduction

Face pareidolia is the tendency to see faces in non-face images such as clouds, houses, or even in a toast slice^1–19^. Face-pareidolia images have the advantage of not displaying facial features such as a mouth or eyes, which inevitably trigger face processing. This makes face-pareidolia (non-face, but face-like) images a useful tool for studying face tuning^2–9,17,20,21^, in particular in clinical populations^3,22^.

Efficient face processing is essential for effective interaction with others and integration into society. Its alterations can lead to maladaptive social interaction or even to social isolation^23,24^. Face processing deficits have been found in individuals with mental disorders, in particular with schizophrenia (SZ)^25,26^. Non-verbal social cognition is reported to be heavily affected in patients with SZ, ranging from deficits in body language reading^27,28^ (for reviews, see refs. ^29,30^) to processing of facial information^31–33^ including inferring emotions and mental states from the eyes^34^. These deficits, in turn, compromise social participation and quality of life^35–38^.

Brain imaging studies on face processing in SZ have predominantly used real-face images. In general, this work reveals impairments in SZ patients compared to typically developing (TD) individuals^39^. Face-specific alterations occur already at early processing stages as indicated by the N170 event-related potential (ERP) component: decreases in N170 amplitude in SZ as compared to TD individuals are face-specific, occurring in response to faces but not houses^40^ or cars^41,42^. Similarly, the face-specific M170 response (neuromagnetic counterpart of N170) localized within the fusiform face area (FFA) is also selectively diminished in SZ^26^.

Do alterations in brain processing in SZ occur when watching face-pareidolia images? Even behavioral (psychophysical) data on face pareidolia in SZ are not only sparse, but rather controversial. Patients with SZ are reported either to identify fewer face-like images as faces^43^ or exhibit higher face-pareidolia scores^44^ than TD individuals. When presented with a set of Arcimboldo-like Face-n-Food images from the least to most resembling a face, SZ patients provided much fewer face responses^45^. Recent work nicely dovetails with this finding demonstrating that lower face-pareidolia rates in SZ are accounted for by a lower visual sensitivity to a face scheme rather than by alterations in cognitive bias^46^. This implies that in SZ, alterations in brain activity in response to face-pareidolia images may occur already at early processing stages.

**Fig. 1:**
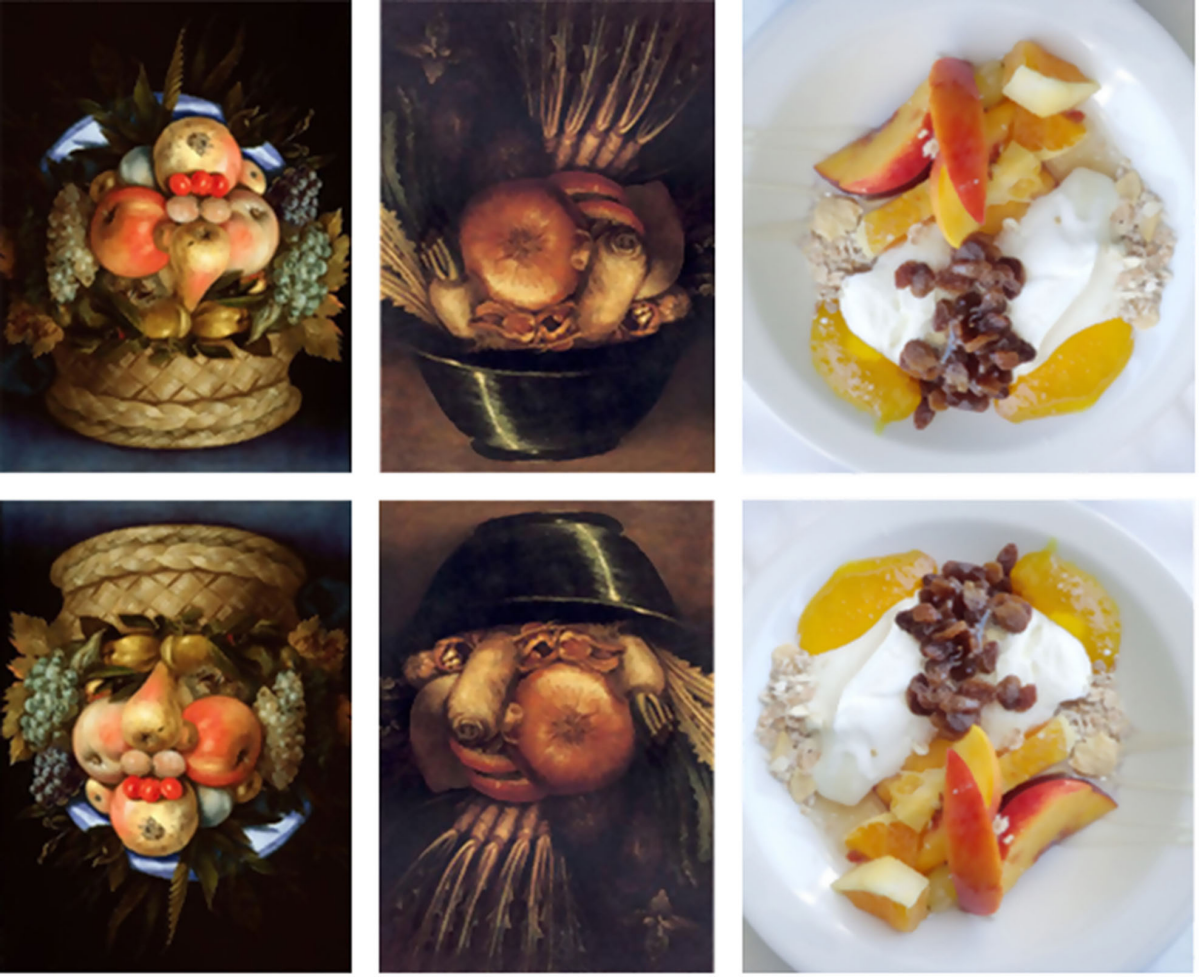
Examples of face-pareidolia images. “Portrait of the man made of fruit” (leftmost) and “The Gardener” (middle; https://commons.wikimedia.org/wiki/Giuseppe_Arcimboldo; public domain) by genius Italian painter Giuseppe Arcimboldo (1526-1593). Rightmost: The least face-resembling Face-n-Food image (from Pavlova et al. Face-n-Food: gender differences in tuning to faces. *PLoS One* **10**, e0130363 (2015), ref.^2^, the Creative Commons Attribution [CC BY] license). Only Face-n-Food images served as stimuli in the present study. The top row represents the images inverted 180° in the image plane.

The present work was directed at uncovering brain communication over time underpinning face-pareidolia impressions in SZ. With this purpose in mind, patients with SZ were administered a task with Arcimboldo-like Face-n-Food images to different degree resembling a face^2–7,9,12,13,45^ (Fig. 1) during simultaneous magnetoencephalographic (MEG) recording of cortical activity. The analysis had been focused on gamma oscillations that underlie not only cognitive and emotional processes such as attention and working memory, but also different components of social cognition such as face processing (including holistic *Gestalt* perception) and body language reading^47–55^. By using the Face-n-Food images, recent work showed that changes in MEG gamma oscillatory activity reflect processing of face-pareidolia images in individuals with major depressive disorder, MDD^13^. In typical development, a cutting-edge MEG analysis of brain connectivity unfolding over time during a task with face-pareidolia Face-n-Thing images reveals mutual feedforward and feedback intra- and interhemispheric communication within and beyond the social brain. In particular, the superior temporal sulcus (STS) and insula (INS) strongly engage in communication with other down- and upstream brain regions either as signal transmitters or recipients^17^.

In this piece of work, we focused on male individuals mainly because SZ is considered a gender (a social construct)/ sex (a neurobiological one)-specific mental disorder with substantial differences in disease manifestation. Males exhibit an earlier age of onset, poorer premorbid social functioning, more severe negative symptoms (especially social withdrawal), higher frequency of substance and alcohol abuse, and higher suicide mortality^56–60^. Males receive diagnosis of SZ 1.1 to 1.4 times more often than females^61–63^. Moreover, individuals with SZ are assumed to exhibit gender-specific profiles in social cognition^38,64,65^.

## Methods

### Participants

Twenty-seven male patients with SZ were recruited mostly from the inpatient (but also outpatient) units at the Department of Psychiatry and Psychotherapy, University Hospital, Eberhard Karls University of Tübingen, Germany. Five patients were excluded due to a very low number of valid responses (due to excessive missing responses and/or responses within the stimulus interval that confounded neuromagnetic traces), which resulted in too few trials available for MEG analysis. One patient failed to follow the instruction. A total of 21 datasets of SZ patients entered final data analysis. The sample size was calculated a priori considering demands of statistical data processing and possible dropouts. Patients of the final sample were aged 32.81 ± 8.09 years (mean ± standard deviation, SD), median, Mdn, 29, 95% confidence interval, CI [29.13; 36.49] (age range, 24–46 years). Most TD male controls participated in our earlier MEG study on face pareidolia in MDD^13^ with the same task and experimental design, and their data were reanalyzed here. In order to attain matching with SZ patients, two additional controls were recruited from the local community. TD individuals were aged 34.10 ± 8.11 years, Mdn, 35, 95% CI [30.40; 37.79] (age range, 21–46 years). No difference in age occurred between SZ and TD individuals (Mann-Whitney test, *U* = 196.5, *p* = 0.560, 2-tailed, n.s.).

Seventeen out of 21 patients were diagnosed with paranoid SZ (ICD-10, F20.0). For four patients, the diagnosis was schizoaffective disorder (ICD-10, F25). The average time from the first diagnosis to examination was 8.06 ± 7.55 years, Mdn, 6.5; 95% CI [4.30; 11.81]; two patients were excluded from this calculation due to missing information. Exclusion criteria comprised preterm birth (<37 gestation weeks) and comorbid neurological and psychiatric conditions such as anxiety disorders, depression, eating disorders, and head injury. Ten out of 21 patients had comorbidities (Table S1, Supplementary material). A history of neurological or mental disorders including SZ, autism spectrum disorders (ASD), MDD, and regular intake of medication served as exclusion criteria for the control group.

The study was conducted in line with the Declaration of Helsinki and approved by the local Ethics Committee at the Medical School, Eberhard Karls University of Tübingen. Informed written consent was obtained from all participants. Participation was voluntary and the data were processed anonymously. Participants received a small monetary compensation for their participation.

### Experimental design and procedure

The task is described in detail elsewhere^13,17^. In brief, the Face-n-Food images (Fig. 1) were presented in either upright or inverted (to 180° in the image plane) orientation. As our previous work^8,13^ revealed a more pronounced impact of display inversion on face pareidolia in males than in females, we focused on male individuals. The other motivation for setting a focus on males was some evidence for the sex differences in gamma oscillatory MEG activity on visual-perceptual tasks^66^. Finally, as mentioned above, we focused on male individuals mainly because SZ is considered a gender-specific mental disorder. Yet, for deeper understanding the brain mechanisms underlying face pareidolia both in typical development and SZ, it is desirable to take a close look at female SZ. During MEG recording, participants were presented with a set of stimuli consisting of 192 trials (12 images × 2 types [original/mirror image] × 2 display orientations [upright/inverted] × 4 repetitions). We prevented a possible adaptation of the visual system to display orientation by limiting the images presentation to up to four images in a row for each orientation. For the same reason, mirror-reversal images were used. By contrast with the most previous neuroimaging work^10,67,68^, but in accord with our own earlier MEG studies^13,17^, participants were directly required to report face pareidolia: In the 2-AFC (two alternative forced-choice) task, on each trial, participants had to indicate by pressing a respective key whether or not they had an impression of a face. Yet, participants were explicitly told that there were no correct and incorrect responses on the task, and they had to rely solely upon their own visual impression. During a pre-recording session consisting of 10 to 15 trials, participants were trained to respond (by pressing respective keys for face and non-face impressions) after stimulus offset to avoid a possible interference of motor responses with the recorded MEG traces. If participants failed to respond, the next trial automatically started after an inter-stimulus interval randomly varying between 3 to 5 s. The stimuli were presented in a pseudo-randomized order for the duration of 1.2 s. Each stimulus subtended a visual angle of 10.2° (with an image size on the screen 12.5 × 12.5 cm at an observation distance of 70 cm). Prior to each image, a small fixation cross appeared in the center of the screen for 2 s. The images were presented via a PROPixx 1,440 Hz DLP LED Projector (VPixx Technologies Inc.; Saint-Bruno, QC, Canada). The visual task was built with the help of Presentation software (Version 20.3, Neurobehavioral Systems, Inc.; Albany, CA, United States). For each participant, the recording session lasted for 12–15 min.

### MEG recording and analysis

MEG measurement was performed at the MEG Center, University Hospital of Tübingen. The recording was conducted in a magnetically shielded chamber (Vacuumschmelze GmbH & Co. KG; Hanau, Germany) with a whole-head MEG system comprising 275 first-order axial magnetic gradiometers (VSM MedTech Inc.; Coquitlam, BC, Canada; for details, see ref.^13^). Neuromagnetic signals were recorded at a rate of 1,171.88 Hz with a 293 Hz antialiasing low-pass filter. Head movement was monitored throughout the entire recording session using three localization coils positioned at the nasion and two (right and left) preauricular sites of the head. Deviation of 6 mm from the initial head position was considered as a cutoff for a valid recording. MEG data analysis was performed with in-house MATLAB scripts (MATLAB 2022a; The MathWorks Inc.; Natick, MA, United States) and the Fieldtrip toolbox (version fieldtrip-20201229^69^).

### Behavioral data analysis

As it was shown in our earlier behavioral work performed with the same images and experimental design^46^, with canonical upright display orientation, SZ patients exhibited deficits in face pareidolia: they provided much fewer face responses than TD controls. Here, a behavioral data analysis was conducted on the preprocessed records (after filtering out artifact-compromised trials) to align them with MEG analyses (see Supplementary Material).

### Data categorization and time-frequency analysis

Similar to ref.^13^, after preprocessing, individual data were classified by Orientation (Upright/Inverted) and Response (Face/Non-face impression). To maximize the contrast between conditions, the present analysis was focused on the face responses when the stimuli were presented with upright orientation and non-face responses when the stimuli were presented inverted 180° in the image plane. This contrast was first calculated for each participant for the time-frequency analysis (time-frequency representation, TFR). To ensure an equal number of trials across conditions, the minimum number of trials (*N*_*m*in_) between the two conditions was calculated and *N*_min_ trials from the other condition for comparison was randomly selected.

On average, 38.62 ± 10.1 trials for SZ patients and 42.33 ± 12.21 trials for TD individuals across conditions and participants entered the final time-frequency analysis with no difference between the groups (*t*(40) = 1.07, *p* = 0.289, 2-tailed, n.s.). Hanning tapers were calculated independently for each condition and each participant in a frequency range from 2 to 98 Hz with a 2 Hz resolution. To minimize spectral leakage of TFR, the time window was fixed at 500 ms with 50–ms sliding windows starting from 1.25 s pre-stimulus to 1.25 s post-stimulus onset^70,71^. Finally, averages across trials were computed, and grand averages of TFR data were calculated.

### Statistical inference

Details on statistical analysis and source reconstruction are provided in refs. ^13,17^. In brief, the TFR data statistics were computed with a 2-tailed cluster-based nonparametric permutation test. The clusters contain 3-dimensional data (spatio-temporo-spectral). The cluster-α was fixed at 0.05 and required a cluster size of minimum 2 neighboring channels. This method corrects for family-wise error (FWE) rates for multiple comparisons^72^. The analyses were confined to the gamma band within the range of 30–90 Hz, with a step size of 5 Hz, in order to enhance the frequency resolution of the targeted frequency band. The significance level of the relative power changes was calculated with respect to baseline (500–300 ms pre-stimulus) for the entire time of stimulus presentation (0–1.2 s) using the Monte Carlo simulation based on a set of 1000 permutations to infer the effects of condition (Face impression/Non-face impression) and group (TD/SZ).

### Source reconstruction

Source localization for the time windows and frequency ranges of significant clusters was performed with a beamformer approach implemented in Fieldtrip^13,69^. Cross-spectral density data were obtained with discrete prolate spheroidal sequence (DPSS) tapers. The spectrum was calculated for the mean of the significant clusters’ frequency range with a time window of 500 ms sliding forward in steps of 50 ms from 1.25 s pre- to 1.25 s post-stimulus. For source reconstruction, a canonical MNI (Montreal Neurological Institute) template specified in a coordinate system defined by the fiducials nasion, left and right preauricular sites was used. A single-shell volume conduction model was derived from the brain scan^73^ and the Polhemus head shape was used for the head model. A regularly spaced grid was established with 1-cm spacing in 3-dimensional space. Source analysis was based on the dynamical imaging of coherent sources (DICS) approach^74^. Common spatial filters for both conditions were then derived from the cross-spectral density (CSD) matrix of the TFR data and leadfield matrix. Applying the spatial common filters resulted in TFRs for each condition and grid point, reflecting the time course of the spectral source strength.

### Connectivity analysis

The connectivity analysis for low and high gamma was performed for frequencies of 40–45 Hz and 65–70 Hz, respectively. In these ranges, the power spectrum of face responses for both SZ patients and TD controls was above the baseline. This analysis assumes that increased coherence is indicative of communication between seeds. Based on our previous work on brain circuits underpinning face pareidolia in TD, the right and left lateral occipital cortex (LOC), along with four other regions engaged in processing of face-pareidolia images^17^ were selected as seed regions in both hemispheres: the insula (INS), superior temporal sulcus (STS), inferior parietal lobe (IPL), and inferior temporal gyrus (ITG). To assess directionality (leading or lagging) of the connections and their potential changes over time (i.e., time course), the phase slope index (PSI) for the connections of the ten seeds with the other seeds was calculated for each participant. The analysis was performed from stimulus onset over the entire stimulus duration in windows of 0.4 s with a 0.1–s overlap (given a total stimulus duration of 1.2 s, overlap of the last time window was 0.2 s). A 2-tailed sign test was performed to assess significance of the connection directionality.

## Results

### Increases in gamma oscillations relative to baseline

In TD individuals, for face responses, early (from 0 to 0.3 s after stimulus onset) significant stimulus-specific (present for face impressions with upright display orientation but absent for non-face impressions with display inversion) increases in gamma oscillatory activity occurred at low frequencies of 30–35 Hz (*p* = 0.037, FWE corrected for multiple comparisons throughout). Yet, increases in the range of 35–40 Hz occurred for both face (*p* = 0.006; 0–0.4 s) and non-face (*p* = 0.025; 0–0.6 s) responses.

**Fig. 2:**
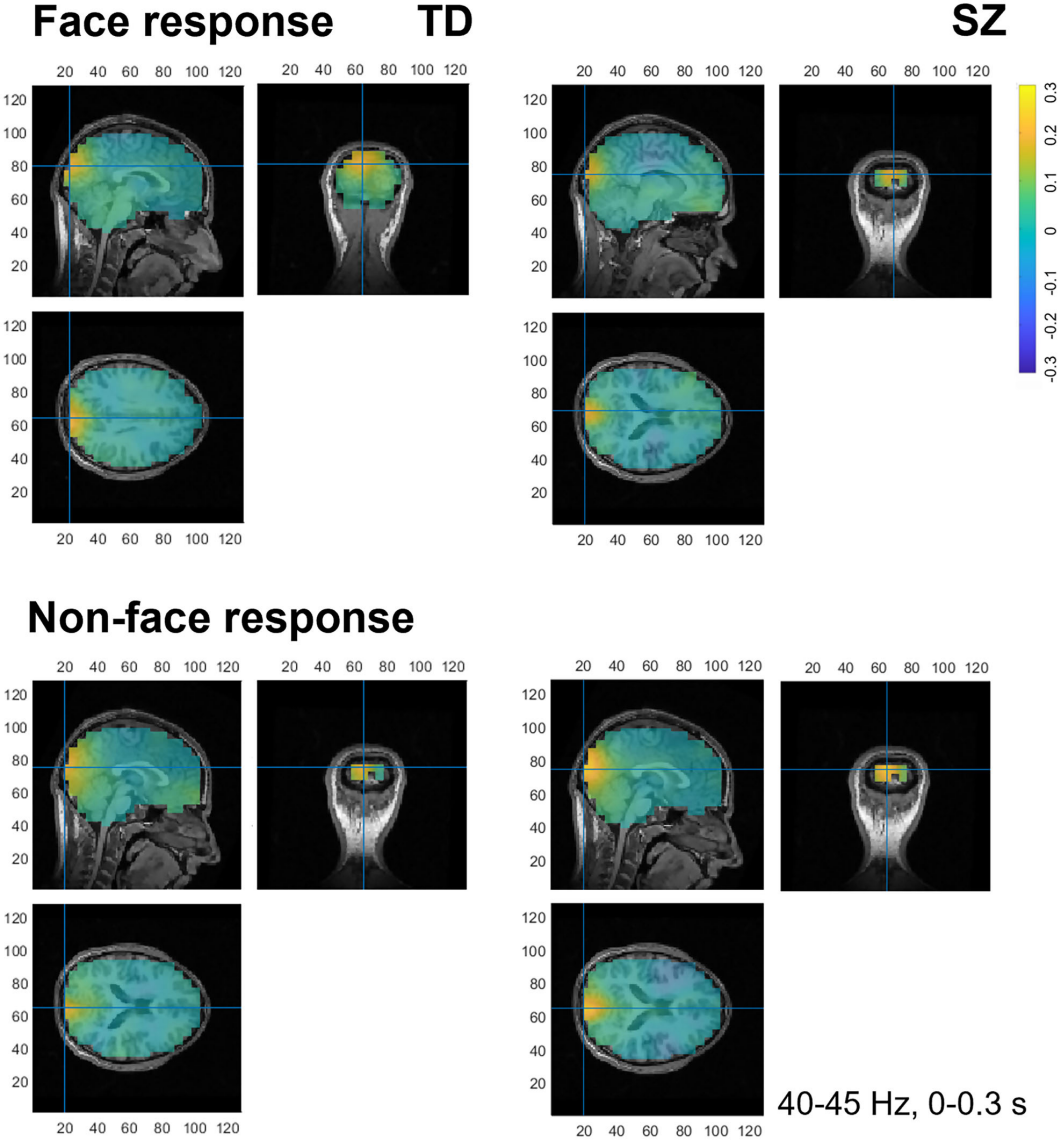
Source reconstruction for face and non-face responses versus baseline in SZ and TD individuals. Early (0–0.3 s) gamma oscillatory activity in the frequency range of 40–45 Hz originated from the MVOC (area of maximum power spectrum) for face responses in SZ (top right panel) and non-face responses in both groups (bottom panels). In TD individuals, the gamma peaks accompanying face response originate with a maximum in the LOC (top left panel). Increases in gamma activity relative to baseline are coded in warm colors.

By contrast, in SZ patients, the peaks at 30–35 Hz and 35–40 Hz were completely absent. Instead, individuals with SZ exhibited early (0–0.3 s) increases for both face (*p* = 0.016) and non-face (*p* = 0.003) responses at a higher frequency of 40–45 Hz. In this frequency range, TD individuals showed bursts in gamma activity for both face and non-face responses (both *p* < 0.001) throughout the entire stimulus duration. As seen in Fig. 2, source localization analysis for the first 0.3 s indicated that for TD individuals, the clusters of increased gamma activity for face responses originated (with a maximum spectrum) from the right LOC, whereas for the non-face responses, they stemmed from the MVOC without pronounced lateralization. In SZ, both for face and non-face responses, increases in the gamma frequency range of 40–45 Hz originated from the MVOC, resembling the increases for non-face responses in TD individuals.

### Between-group contrast for face pareidolia

**Fig. 3:**
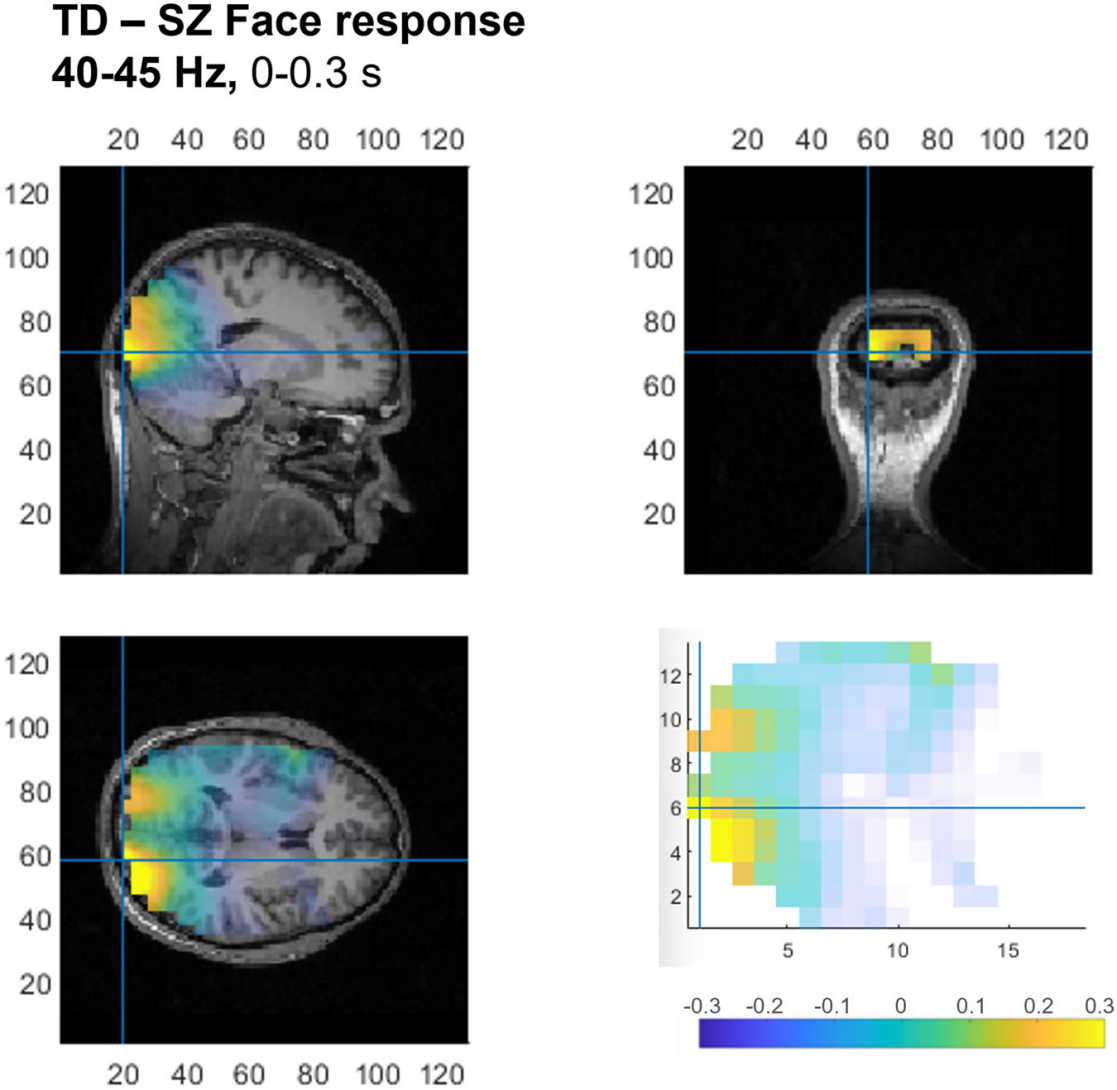
Source reconstruction for contrast in gamma activity (40-45 Hz) for face responses between TD and SZ individuals. Early (0–0.3 s) maximum group differences occur in the right LOC. Increases in gamma activity in TD as compared to SZ individuals are coded in warm colors. Top panel along with bottom left image represent voxel-level source activity interpolated onto an MRI scan, right bottom image shows voxel-level activity at lower spatial resolution with maximum spectrum power indicated by blue cross.

When contrasting gamma oscillatory activity for face responses between TD and SZ individuals, significantly greater activity in TD was found during the whole stimulus duration for the frequency bands of 30–35 Hz (*p* = 0.005) and 35–40 Hz (*p* = 0.003), from 0.05 to 1.2 s for the frequency band of 40–45 Hz (*p* = 0.011), and from 0.01 to 0.6 s for the frequency band of 55–60 Hz (*p* = 0.034). For 65–70 Hz, peaks in gamma activity tended to be significant (*p* = 0.08) for the whole stimulus duration. As seen in Fig. 3, the maximum TD-SZ contrast for the frequency band 40–45 Hz during the first 0.3 s originated from the right LOC.

### Differences in brain communication between SZ and TD individuals

**Fig. 4:**
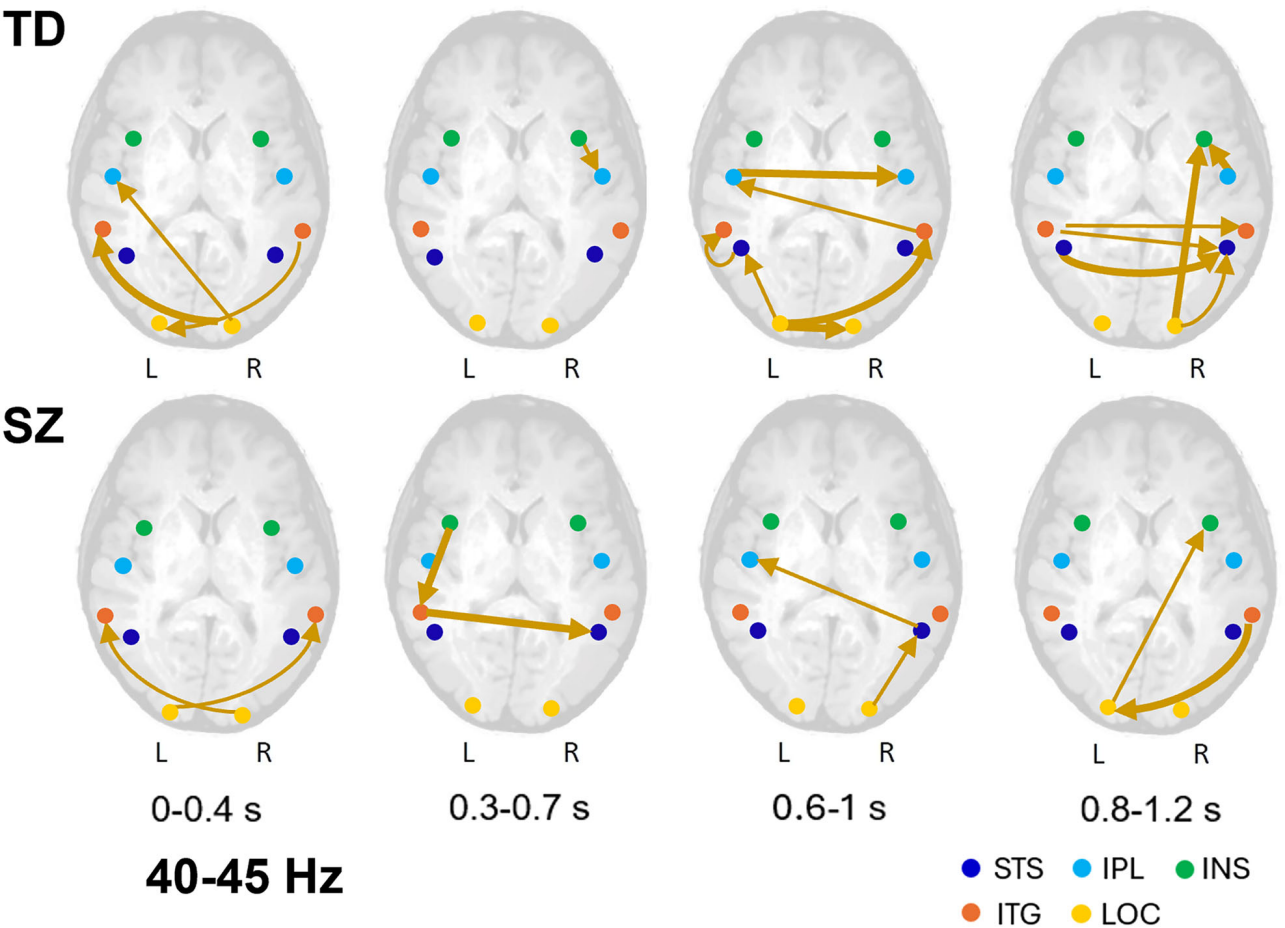
Schematic representation of functional connectivity unfolding over time between the brain areas at gamma frequences of 40–45 Hz for images triggering face pareidolia. Significant connections superimposed on the schematic brain are shown by thick arrows (*p* < 0.05), while thin arrows represent connections that tend to be significant (0.05 < *p* < 0.08). Colored blobs indicate the regions of interest: STS superior temporal sulcus, IPL inferior parietal lobule, INS insula, ITG inferior temporal gyrus, LOC lateral occipital cortex. TD individuals are represented in the top row and SZ patients in the bottom row.

**Fig. 5:**
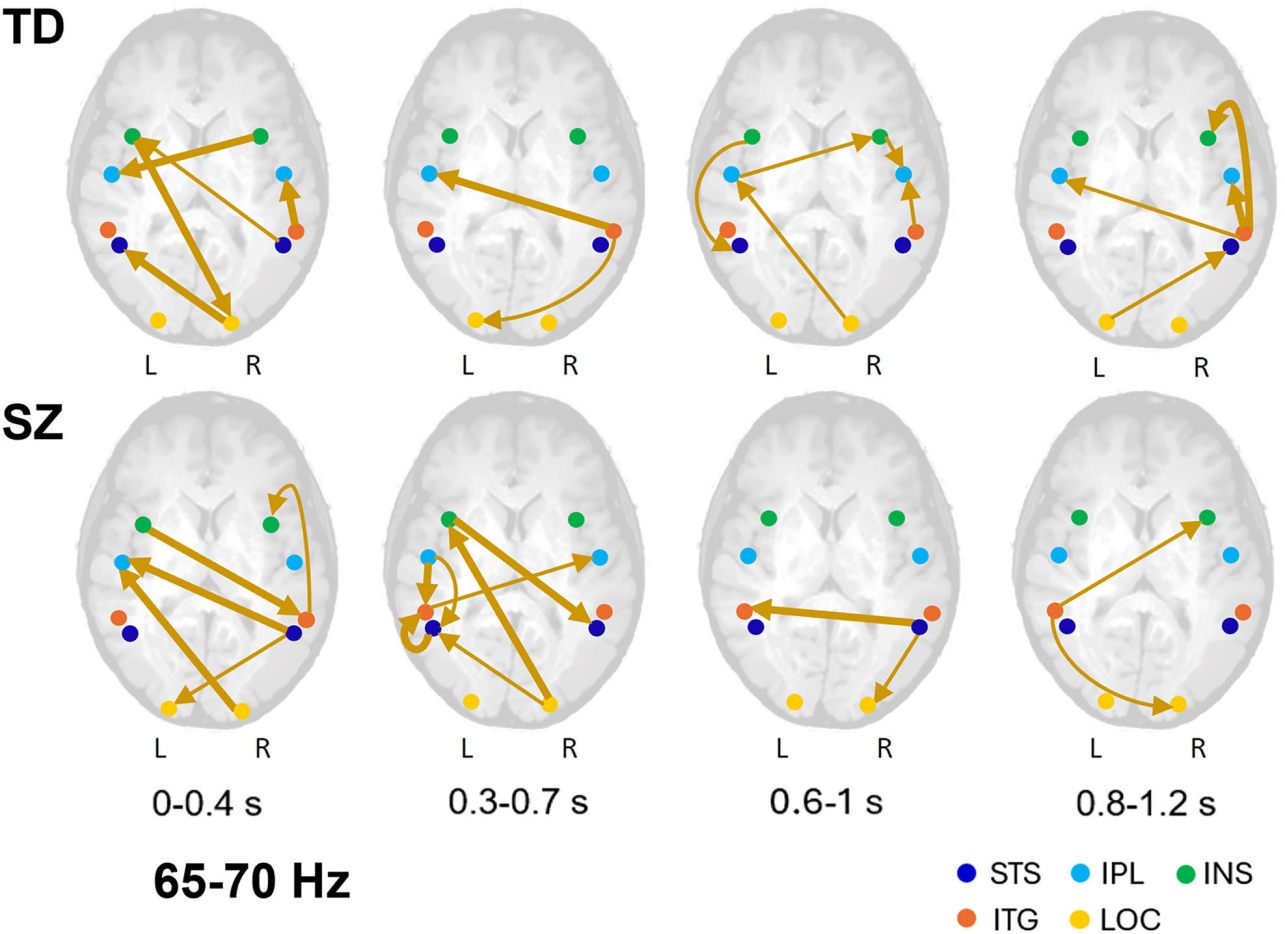
Schematic representation of functional connectivity unfolding over time between the brain areas at gamma frequences of 65–70 Hz for images triggering face pareidolia. Significant connections superimposed on the schematic brain are shown by thick arrows (*p* < 0.05), while thin arrows represent connections that tend to be significant (0.05 < *p* < 0.08). Colored blobs indicate the regions of interest: STS superior temporal sulcus, IPL inferior parietal lobule, INS insula, ITG inferior temporal gyrus, LOC lateral occipital cortex. Brain communication in TD individuals is represented in the top row, and SZ patients in the bottom row.

The outcome of the brain connectivity analysis unfolding over time separately for the low (40-45 Hz) and high (65–70 Hz) gamma activity between the LOC in both hemispheres and the key areas of the social brain is represented in Figs. 4 and 5. Detailed description is provided in Supplementary Material. In the low gamma range of 40–45 Hz (Fig. 4), in the first time window (0–0.4 s), in both SZ and TD individuals, low-level visual processing areas actively engage in brain communication. In SZ, interaction of the LOC of both hemispheres is primarily limited to sending signals to the ITG of the opposite hemisphere. In TD, in addition to communication between the right LOC (which is a source of gamma oscillations accompanying face pareidolia; see above) and the left ITG, the right LOC transmits signals to the IPL of the left hemisphere, and the left LOC receives feedback from the higher-order regions such as the right ITG. Later (0.3–0.7 s), in TD, the brain is almost silent with only the right INS transmitting signals to the IPL of the same hemisphere. By contrast, in SZ, the key areas of the social brain come into a play with feedback communication between the left INS transmitting signals to the ITG of the same hemisphere, and then to the right STS, a pivot area of the social brain.

At later latencies (0.6–1 s and 0.8–1.2 s), in both SZ and TD, the LOC actively communicates with the social brain, albeit in TD, this interaction is more intense than in SZ. In SZ, in particular in the last time window, only the right ITG transmits information to the left LOC that in turn sends feedforward signals to the right INS. In TD, the right INS receives signals from the LOC and IPL of the same hemisphere, and the right STS from the left STS, right LOC, and the left ITG. In addition, the left ITG sends messages to the ITG of the opposite hemisphere.

In the high gamma range (65-70 Hz, Fig. 5), in the first time window (0-0.4 s), the LOCs in both hemispheres actively (but with diverse patterns in SZ and TD individuals) communicate with the social brain. In TD, the right LOC transmits signals to the STS of the opposite hemisphere and receives feedback information from the left INS; other social brain areas also interact with each other. In SZ, the right LOC transmits signals to the IPL of the opposite hemisphere, whereas the right STS also sends messages to the left IPL as well as to the left LOC. Later in time (0.3–0.7 s), communication in TD is limited mainly to transmission of signals from the right ITG to the INS and LOC of the left hemisphere, while in SZ, it remains intense exhibiting a few loops: for example, the right LOC transmits signals to the left STS, which in turn sends information to the left ITG and receives signals from the left IPL, which also transmits information to the left ITG (Fig. 5). In the last time windows, brain communication in SZ is rather limited to transmission of signals from the right STS to the right LOC and left ITG (0.6–1 s) and from the left ITG to the right INS and LOC (0.8–1.2 s). In TD, brain communication is richer than in SZ with engagement of a number of the social brain areas. For example, the right LOC transmits signals to the left IPL, then the left IPL to the right INS, and the right INS to the right IPL, which also receives information from the right ITG.

Overall, dynamic brain communication in the low (40–45 Hz) and high (65–70 Hz) gamma ranges differs at earlier stages of processing of face-pareidolia images. In the gamma range of 65–70 Hz, brain communication is more intense between the LOC and the social brain areas (such as the INS and STS).

In the low gamma band (40–45 Hz), in SZ, early brain communication is primarily limited to transmitting signals from the right and left LOCs to the ITGs of the opposite hemispheres, whereas in TD, the right LOC, a vital source of gamma oscillations supporting face pareidolia, transmits signals not only to the left ITG, but also to the left IPL. Already at this stage, the left LOC is engaged in feedback communication as a recipient of signals from the right ITG.

In the high gamma band (65–70 Hz), both in SZ and TD, early brain communication between the LOCs and the social brain is already rather intense. However, in TD, the right LOC not only transmits signals to the left STS but also serves as a recipient of signals from the left INS, which in turn, receives messages from the right STS, a key node of the social brain. In SZ, however, engagement of the right LOC is limited to sending messages to the right IPL.

## Discussion

This work was aimed at investigation of brain communication unfolding over time that underwrites face pareidolia in male SZ. For this purpose, we presented SZ and TD individuals with a set of Arcimboldo-like Face-n-Food images while simultaneously recording MEG activity. The outcome shows: (i) Already at early processing stages, the bursts of gamma oscillatory activity differ between SZ and TD individuals in the frequency range and underlying topography. In TD individuals, gamma peaks for face pareidolia occur at low frequencies of 30–35 Hz, whereas in SZ, they take place starting from higher frequencies of 40–45 Hz. In this range, in TD, the burst accompanying face-pareidolia impressions originates from the right LOC, whereas for non-face impressions, the peak shows the maximum in the MVOC. In SZ, both for face-pareidolia and non-face impressions, the peaks’ maximum originate from the MVOC without clear hemispheric lateralization. (ii) When contrasting gamma activity for face-pareidolia responses between TD and SZ individuals, the maximum difference in oscillatory activity for frequencies 40–45 Hz during the first 0.3 s from stimulus onset originates from the right LOC. (iii) Overall, in SZ, early communication of the right LOC, a source of gamma oscillations supporting face pareidolia, is primarily limited to unidirectional feedforward transmission of signals, whereas in TD, this area also serves as a recipient of sophisticated feedback communication from the social brain.

### Alterations in early low gamma activity in schizophrenia

This work focuses on alterations in gamma oscillatory activity, which has been associated with face processing both in the neurotypical population^13,17,75,76^ and SZ^77,78^. TD individuals exhibit early increases relative to baseline in response to face-pareidolia images at low gamma frequencies (30–40 Hz), while in SZ patients, the peaks first occur only at higher frequencies (40–45 Hz). Most important, in terms of brain topography, as seen in Fig. 2, for the frequency range of 40–45 Hz, in TD individuals, the burst accompanying face-pareidolia impressions was observed in the right LOC whereas for non-face responses, the peak attained its maximum in the MVOC without clear hemispheric lateralization.

The LOC and MVOC seem to perform different functions during image and object recognition while interacting with each other^79–81^. The medial regions comprising the lingual gyrus (LG) and collateral sulcus (CoS) are primarily involved in surface and texture discrimination, while the LOC dwells with shape recognition^79,81^. Further support for this dissociation is provided by lesion studies. Patients with damage to the LOC are unable to perceive form and shape of objects (visual form agnosia), while they can perceive their texture and color^82^. By contrast, patients with damage to the MVOC are unable to perceive color but can still perceive shape (cerebral achromatopsia^83^).

The present work emphasizes the functional role of the LOC for face pareidolia. The LOC seems to be a vital source of gamma oscillations likely playing a key role in the perception of face-pareidolia images as a whole (*Gestalt*) rather than simply a number of food elements unrelated to each other. Yet, for SZ patients, both for face-pareidolia and non-face impressions, the peaks of gamma oscillations originate from the MVOC. The functional role of the right LOC for face pareidolia is further confirmed by the contrast in gamma oscillations (40–45 Hz) between TD individuals and SZ patients, which shows a maximum difference in this area (Fig. 3).

Our previous behavioral work demonstrates that face-pareidolia images elicit fewer face impressions in SZ patients as compared to healthy person-by-person matched controls^45^. Moreover, reduced face-pareidolia rates in SZ are accounted for by a lower visual sensitivity rather than by changes in cognitive bias^46^, suggesting that in SZ, alterations in brain activity in response to face-pareidolia images may occur already at rather early processing stages. The present MEG findings nicely dovetail with behavioral outcome, providing a neurobiological basis for understanding the origins of face-pareidolia deficits in SZ.

### Functional role of low and high gamma oscillations in face pareidolia

Converging evidence from electrophysiological, physiological and anatomical studies suggests that abnormalities in the synchronized oscillatory activity of neurons may have a central role in the pathophysiology of schizophrenia^84^. Low gamma oscillations (30–60 Hz) are typically associated with sensory processing, *Gestalt* perception, and attentional resources^85,86^. Alterations in low gamma activity accompanying face pareidolia in SZ patients may reflect differences in processing of face-pareidolia images already at a rather basic level. Such alterations may impair early perceptual-cognitive integration^84,87^, which is essential for efficient processing of both real faces and face-pareidolia images^88,89^.

By contrast, high gamma activity (>60 Hz) is considered to be related to complex cognitive processing and higher cognitive functions^85,86^. Patients with SZ were previously shown to exhibit reduced high gamma oscillations (60–120 Hz) in response to illusory Mooney faces along with elevated response time and reduced detection rates as compared to controls^90^. In the present study, however, no differences in the power of high gamma activity were found between SZ and TD individuals in response to face-pareidolia images. This at least partly dovetails with our previous behavioral findings indicating the lack of differences between SZ and TD in cognitive bias/decision criteria in response to face-pareidolia images^46^. Yet, as revealed by the present brain connectivity analysis unfolding over time, a distinct pattern is observed in SZ as compared to TD individuals for the high gamma frequency of 65–70 Hz. This will be discussed in more detail below.

### Structural brain differences in schizophrenia related to face processing

Neuroimaging has identified abnormalities in the structure, function, and connectivity of multiple brain areas in patients with SZ, which are associated with social-cognitive deficits^91,92^. Are the social brain areas that may be engaged in face pareidolia affected in SZ?

The STS, a key node of the social brain^93–96^, and the INS are associated with emotional salience and social processes^97–99^ and repeatedly reported to be involved in face pareidolia^10,17,100,101^. The INS communicates with multiple brain networks and regions, including the medial prefrontal cortex, amygdala, somatosensory cortices, and the brainstem nucleus of the solitary tract, facilitating the integration of sensory, emotional, and cognitive information^99^. Abnormalities in the INS structure, including reductions in grey matter and cortical thinning, are suggested to serve as a biomarker for susceptibility to SZ^92^. In SZ, decreased fMRI response of the INS is reported during presentation of neutral faces^102^. In general, this relatively small brain structure is considered to be underestimated in clinical neuroscience, neurology, and psychiatry^103,104^.

A reduced gray matter volume in SZ was also found bilaterally in the ITG^105^, a structure engaged (among other functions) in representation of faces^106,107^. In agreement with this, whole-brain fMRI in awake rhesus macaques shows that the ITG and STS exhibit selectivity for face-pareidolia images as compared to non-face objects^108^. A male patient LH suffering brain damage to the right ITG after an accident^109,110^ was unable (upon recovery) to recognize faces or discriminate between them, or even recognize faces that were familiar to him before the accident and had been diagnosed with face prosopagnosia. In daily life, LH (and other patients with similar brain damage) use non-facial cues, such as height, hair color, and voice to differentiate between people. In addition, the ITG may also play a key role in semantic memory^111^ and, therefore, in naming of face-pareidolia images.

The IPL is bilaterally involved in social cognition and emotion perception, but with stronger right hemispheric engagement^112,113^. The IPL has been also linked to declarative memory retrieval, with the left IPL associated with semantic aspects, and the right IPL with perceptual aspects of memory^112^. In SZ, a gender-specific gray matter decline was found in this area as well: male patients showed a 6% reduction in gray matter volume and a reversal of normal left-greater-than-right asymmetry in the IPL, while female patients exhibited only a 2% reduction and no significant volume or asymmetry differences compared to healthy females^114,115^. The present study indicates altered patterns of communication between these areas of the social brain in males with SZ, in particular, in the high gamma range of 65-70 Hz (Fig. 5).

### Brain-communication alterations in schizophrenia

An advanced brain-connectivity analysis unfolding over time was performed at low (40–45 Hz, Fig. 4) and high gamma frequencies (65–70 Hz; Fig. 5) between the LOC and several areas of the social brain (such as the STS, INS, and IPL). These areas had been shown to play a central role in face pareidolia in healthy individuals^17^.

At low gamma frequencies (40–45 Hz), in SZ, early brain communication is unidirectional, being primarily limited to transmitting signals from the LOCs to the ITGs of the opposite hemispheres. At high gamma frequencies (65–70 Hz), both in SZ and TD individuals, early brain communication between the LOC and the social brain is already rather intense. However, in TD, the right LOC not only transmits signals to the left STS, but also serves as a recipient of feedback signals from the left INS, which in turn, receives messages from the right STS, a key node of the social brain. In SZ, however, engagement of the right LOC remains unidirectional and is limited to sending feedforward messages to the higher-order brain regions.

Later, at both low and high gamma frequencies, brain communication in TD becomes almost silent, suggesting processing of face-pareidolia images is mainly completed and the brain communication *takes a break*, in the sense that the brain does not need a high degree of synchronization. At later stages, however, brain communication intensifies again with mutual feedforward and feedback intra- and interhemispheric interaction. In particular, the hubs of the social brain, the STS and INS of the right hemisphere, strongly engage in communication either as signal transmitters or recipients. Presumably, this communication underwrites adjustment of face-pareidolia interpretations, additional associations with images, and reflects modifications in inferring of emotional and social information. By contrast, in SZ, brain communication is less intense.

The present findings align with the broader view that schizophrenia involves network-level dysconnectivity, especially within circuits supporting social cognition^116^. In a nutshell, the outcome of the present study provides a blueprint for further investigation of face pareidolia in mental, neurological, and neurodevelopmental disorders, predominantly those characterized by social cognitive deficits, and a skewed gender/sex ratio. In particular, a closer look at brain communication including additional regions commonly known to be engaged in processing of real face as well as face-pareidolia images^100,117^, such as the amygdala and fusiform gyrus^118,119^, could offer a clearer understanding of the neural communication underlying face pareidolia and its alteration in SZ and other mental disorders. Additionally, future examination of face pareidolia and underlying brain communication in female SZ would ensure a more comprehensive view on the origins of possible gender/sex specificity as well as alterations in social cognition in SZ at large.

## Supplementary information

Supplementary Material

## Data Availability

All data are available in the manuscript and/or Supplementary Material.
