# Supplementary material for: Dynamic brain communication underlying face pareidolia in male schizophrenia
Source: Schizophrenia (Heidelb). 2025 Aug 13;11(1):112. doi: 10.1038/s41537-025-00656-4 (PMC12350684; doi:10.1038/s41537-025-00656-4)
Supplement: Supplementary file 1 — Supplementary Material [file 41537_2025_656_MOESM1_ESM.pdf]

SUPPLEMENTARY MATERIAL

DYNAMIC BRAIN COMMUNICATION UNDERLYING FACE PAREIDOLIA IN MALE SCHIZOPHRENIA

Valentina Romagnano[1], Julian Kubon[1], Alexander N. Sokolov[1], Andreas J. Fallgatter[1,2], Christoph Braun[3,4], Marina A. Pavlova[1,*]

[1] Department of Psychiatry and Psychotherapy, Tübingen Center for Mental Health (TüCMH), Medical School and University Hospital, Eberhard Karls University of Tübingen, Tübingen, Germany

[2] German Center for Mental Health (DZPG), Partner Site Tübingen, Germany

[3] MEG Center, Medical School and University Hospital, Eberhard Karls University of Tübingen, Tübingen, Germany

[4] Hertie Institute for Clinical Brain Research, Medical School and University Hospital, Eberhard Karls University of Tübingen, Tübingen, Germany

SUPPLEMENTARY PARTICIPANT INFORMATION

Table S1. Comorbidity in SZ patients.

	**Comorbidities (ICD-10 Code)**
P01	Cannabis dependence syndrome (F12.2)
P02	Cannabis dependence syndrome (F12.2)
P03	Attention-deficit hyperactivity disorder (F90.0)<br>Harmful use of multiple drugs and other psychoactive substances (F19.1)<br>Use of other stimulants, including caffeine dependence syndrome (F15.2)<br>Cannabis dependence syndrome (F12.2)<br>Alcohol dependence syndrome (F10.2)<br>Cocaine dependence syndrome (F14.2)
P05	Obsessive-compulsive disorder (F42.0)<br>Mixed and other personality disorders (F61)<br>Dependence syndrome due to tobacco (F17.2)
P08	Post-traumatic stress disorder (F43.1)
P13	Multiple substance dependence disorder (F19.10)
P14	Emotionally unstable personality disorder (F60.31)<br>Cannabis dependence syndrome (F12.2)
P18	Dependence syndrome due to multiple drugs and other psychoactive substances (F19.2)
P19	Harmful use of multiple drugs and other psychoactive substances (F19.1)
P21	Tobacco dependence (F17.2)<br>Harmful use of cannabis (F12.1)<br>Harmful use of alcohol (F10.1)<br>Harmful use of other stimulants (F15.1)

All patients were under routine medical drug treatments: antipsychotics [olanzapine (5 individuals), quetiapine (5), risperidone (7), aripiprazole (6), haloperidol (1), clozapine (3), and

amisulpride (2)], sedatives [lorazepam (2), promethazine (1)], antidepressants [citalopram (2), bupropion (1), mirtazapine (1), and sertraline (1)], mood stabilizer [lithium (2)], and psychostimulant [methylphenidate (1)].

SUPPLEMENTARY METHODS

Normality of data distribution

All data sets were routinely checked for normality of distribution with the Shapiro-Wilk test. For normally distributed data, we further used parametric statistics. Nonparametric statistical tests were computed for non-normally distributed data and, in addition to means and standard deviations (SDs), medians (Mdns) and 95% confidence intervals (CIs) are reported. Statistical testing of behavioral data was performed with MATLAB (version 2022a; MathWorks, Inc., Natick, Ma, USA; Gibbons and Chakraborti, 2010) and JMP (version 16.2; SAS Institute, Cary, NC, USA) packages.

Data preprocessing

For each participant, MEG data were segmented in 192 trials lasting from 1.5 s before through 1.5 s after stimulus onset and covering the entire stimulus presentation of 1.2 s. In this way, filter artifacts were excluded at the beginning and at the end of the segments. The data were high-pass filtered at 1 Hz. A fixation cross preceded the stimulus onset for 2 s. The time between 0.5 and 0.3 s prior to stimulus presentation was chosen as the baseline. The data were first visually inspected for artifacts to exclude channels (no more than 15 per participant out of a total of 275 channels) and/or trials that exhibited substantial variance across channels (exceeding $> 3 \times 10^{-25}$ T^2/Hz). The channels left out were interpolated for sensor-level analyses. Data were then downsampled to 250 Hz and underwent independent component

analysis (ICA) for additional artifact rejection. From 272 components resulting from data decomposition, the first 70 were visually inspected for ocular, muscular, and cardiac artifacts. The components and/or trials with the artifacts were discarded. The final dataset included a total of 3,594 trials for patients and 3,601 trials for controls with no significant difference between the groups (Mann-Whitney test, $U = 213.5$, $p = 0.873$, 2-tailed, n.s.).

SUPPLEMENTARY RESULTS

Connectivity analysis for low gamma frequencies of 40-45 Hz

Analysis of brain connectivity unfolding over time for the low gamma frequency of 40-45 Hz reveals feedforward and feedback communication between the LOC and the social brain (**Fig. 4**, main text). At early latencies (0-0.4 s), brain communication in both groups involves primarily the LOC and ITG bilaterally. In SZ patients, this communication is unidirectional: both the LOC-L and LOC-R transmit information to the ITG of the opposite hemisphere (ITG-R: mean connection strength as assessed by the phase slop index, PSI, 0.036±0.097, p = 0.078; ITG-L: 0.043±0.115, p = 0.078). In TD controls, however, the LOC-R transmits information to both the ITG-L (0.061±0.120, p = 0.027) and IPL-L (0.033±0.084, p = 0.078), while the LOC-L receives feedback information from the higher-order ITG-R (0.057±0.115, p = 0.078). That is, already at early latencies, the information flow in TD individuals is bidirectional.

Later on, within 0.3-0.7 s, in SZ patients, the key areas of the social brain become active, exhibiting rather strong feedback communication: the INS-L sends signals to ITG-L (0.058±0.125, p = 0.027) that in turn transfers them further to the STS-R (0.05±0.137, p = 0.027). By contrast, in TD controls, the brain is almost silent, with communication limited to signal transfer from the INS-R to IPL of the same hemisphere (0.079±0.161, p = 0.078).

At later latencies (0.6-1 s), in both SZ and TD individuals, the LOCs again interact with the social brain, primarily with the areas of the right hemisphere. Specifically, in SZ, the LOC-R sends signals to the STS-R (0.042±0.127, $p = 0.078$), which are further transferred to the IPL-L (0.02±0.108, $p = 0.078$). In TD controls, however, the LOC-L sends out multiple signals to the LOC-R (0.037±0.111, $p = 0.027$), ITG-R (0.062±0.091, $p = 0.027$), and STS-L (0.069±0.148, $p = 0.078$), which in turn transmits information to the ITG-L (0.037±0.168, $p = 0.078$). The ITG of the opposite hemisphere (i.e., ITG-R) transmits signals to the IPL-L (0.041±0.14, $p = 0.078$), which conveys them back to the IPL-R (0.041±0.162, $p = 0.007$).

Finally, in the time window of 0.8-1.2 s, in SZ patients, the LOC-L receives feedback information from the ITG-R (0.048±0.102, $p = 0.027$) and sends signals to the INS-R (0.039±0.106, $p = 0.078$). In TD, communication is again much more intense. The LOC-R sends signals both to the STS-R (0.038±0.078, $p = 0.078$) and INS-R (0.078±0.117, $p = 0.027$), which also receives signals from the IPL-R (0.06±0.124, $p = 0.027$). The STS-R also receives signals from both the STS-L (0.02±0.16, $p = 0.027$) and ITG-L (0.038±0.114, $p = 0.078$). In addition, the ITG-L transmits signals to the ITG-R (0.021±0.095, $p = 0.078$).

Connectivity analysis for high gamma frequencies of 65-75 Hz

Analysis of brain connectivity unfolding over time for the high gamma frequency of 65-70 Hz reveals intense early (0-0.4 s) communication in both TD and SZ individuals, with multiple, mostly interhemispheric connections between the visual and social-brain areas (**Fig. 5**, main text). In TD controls, the ITG-R sends signals to the IPL-R (0.060±0.125, $p = 0.027$), the IPL-L receives signals from the INS-R (0.036±0.096, $p = 0.027$). The INS-L receives signals from the STS-R (0.052±0.094, $p = 0.078$) and sends feedback to the LOC-R (0.047±0.113, $p = 0.007$), which also communicates with the STS-L (0.072±0.111, $p = 0.027$). In SZ patients, the ITG-R receives signals from the INS-L (0.032±0.095, $p = 0.027$) and transmits them further to

the INS-R (0.062±0.127, $p = 0.078$). The STS-R sends multiple signals to the left hemisphere, namely, both the LOC-L (0.007±0.1, $p = 0.078$) and IPL-L (0.059±0.126, $p = 0.027$). The IPL-L also receives signals from the LOC-R (0.037±0.118, $p = 0.027$).

Later on, at latencies of 0.3-0.7 s, while the communication remains intense in SZ patients, in TD individuals it becomes restrained with only two interhemispheric connections: the ITG-R sends signals to both the IPL-L (0.040±0.079, $p = 0.007$) and LOC-L (0.034±0.106, $p = 0.078$). In SZ patients, the LOC-R sends signals to both the STS-L (0.008±0.129, $p = 0.078$) and INS-L (0.039±0.093, $p = 0.027$), which in turn communicates with the STS-R (0.042±0.136, $p = 0.027$). The STS-L sends signals to the ITG-L (0.043±0.11, $p = 0.007$) and receives signals from the IPL-L (0.061±0.111, $p = 0.078$). The IPL-L sends signals to the ITG-L (0.056±0.059, $p = 0.027$), which also sends signals to the IPL-R (0.059±0.103, $p = 0.078$).

During the second half of stimulus presentation, brain communication in TD individuals becomes again intense. Between 0.6 and 1 s, the INS-L sends signals to the STS-L (0.055±0.172, $p = 0.078$). In addition, the LOC-R tends to transmit information to IPL-L (0.041±0.70, $p = 0.078$), which sends it further to the INS-R (0.057±0.088, $p = 0.\ 078$). The IPL-R receives signals both from the INS-R (0.055±0.145, $p = 0.078$) and ITG-R (0.061±0.096, $p = 0.078$). In SZ patients, the STS-R transmits signals to both the LOC-R (0.057±0.115, $p = 0.078$) and ITG-L (0.039±0.068, $p = 0.026$).

In the last time window of 0.8-1.2 s, the ITG-R is particularly active in TD individuals, sending signals to the IPL of both hemispheres (IPL-L: 0.017±0.136, $p = 0.078$; IPL-R: 0.062±0.117, $p = 0.027$) and INS-R (0.054±0.114, $p = 0.027$). The LOC-L tends to transmit signals to the STS-R (0.038±0.123, $p = 0.078$). In SZ, brain communication is rather restricted, with the ITG-L sending signals to the INS-R (0.061±0.107, $p = 0.078$) and LOC-R (0.002±0.14, $p = 0.078$).

Behavioral outcome

As indicated in the main text, earlier behavioral work performed with similar face-pareidolia images and the same experimental design indicated with canonical upright display orientation, SZ patients exhibited deficits in face pareidolia: they provided much fewer face responses than TD controls (Romagnano et al., 2022). Here, the outcome of a behavioral data analysis is reported that was conducted on the preprocessed records (after filtering out artifact-compromised trials) to align them with MEG analyses. A 2-way mixed-model ANOVA was performed on individual face response rates with the between-subject factor Group (SZ/TD) and within-subject factor Display Orientation (Upright/Inverted). As expected, a main effect of Display Orientation was significant ($F(1,82) = 51.19$, $p < 0.001$, effect size, $\eta^2 = 0.56$) with lower face response rates for the inverted stimuli. A main effect of Group ($F(1,82) = 0.045$, $p = 0.836$, n.s.) and an interaction between these factors ($F(1,82) = 0.36$, $p = 0.552$, n.s.) were not significant. Post-hoc analysis indicated that (*i*) for both upright (SZ: 0.545±0.135, TD: 0.565±0.121; $t(40) = 0.57$, $p = 0.940$, n.s., 2-tailed Tukey honestly significant difference, HSD tests throughout, corrected for multiplicity) and inverted display orientation (SZ: 0.381±0.199; TD: 0.371±0.186; $t(40) = 0.28$, $p = 0.993$, n.s.), no significant differences in face response rates occurred between the SZ and TD groups while (*ii*) display inversion substantially impeded face recognition in SZ ($t(20) = 4.63$, $p = 0.0002$, effect size, Cohen's $d_{RM} = 1.44$) as well as in TD individuals ($t(20) = 5.48$, $p < 0.0001$, $d_{RM} = 1.51$).

Within-group comparisons of face-pareidolia responses with upright orientation with non-face responses in inversion (i.e., the conditions taken for the MEG analysis) showed no significant differences between the conditions in both groups (SZ, $t(40) = 0.51$, $p = 0.615$, n.s.; TD, $t(40) = 0.76$, $p = 0.456$, n.s.).

References

Gibbons, J.D. & Chakraborti, S. *Nonparametric Statistical Inference*, 5th edition. (CRC Press, 2010).

Romagnano, V., Sokolov, A.N., Steinwand, P., Fallgatter, A.J. & Pavlova, M.A. Face pareidolia in male schizophrenia. *Schizophrenia* **8**, 112 (2022).
